# Tailoring Adjuvant Endocrine Therapy for Postmenopausal Breast Cancer: A CYP2D6 Multiple-Genotype-Based Modeling Analysis and Validation

**DOI:** 10.1371/journal.pone.0015649

**Published:** 2010-12-20

**Authors:** Ke-Da Yu, A-Ji Huang, Zhi-Ming Shao

**Affiliations:** Department of Oncology, Shanghai Medical College, Cancer Institute and Cancer Center, Institutes of Biomedical Science, Fudan University, Shanghai, People's Republic of China; Massachusetts General Hospital, United States of America

## Abstract

**Purpose:**

Previous studies have suggested that postmenopausal women with breast cancer who present with wild-type *CYP2D6* may actually have similar or superior recurrence-free survival outcomes when given tamoxifen in place of aromatase inhibitors (AIs). The present study established a *CYP2D6* multiple-genotype-based model to determine the optimal endocrine therapy for patients harboring wild-type *CYP2D6*.

**Methods:**

We created a Markov model to determine whether tamoxifen or AIs maximized 5-year disease-free survival (DFS) for extensive metabolizer (EM) patients using annual hazard ratio (HR) data from the BIG 1-98 trial. We then replicated the model by evaluating 9-year event-free survival (EFS) using HR data from the ATAC trial. In addition, we employed two-way sensitivity analyses to explore the impact of HR of decreased-metabolizer (DM) and its frequency on survival by studying a range of estimates.

**Results:**

The 5-year DFS of tamoxifen-treated EM patients was 83.3%, which is similar to that of genotypically unselected patients who received an AI (83.7%). In the validation study, we further demonstrated that the 9-year EFS of tamoxifen-treated EM patients was 81.4%, which is higher than that of genotypically unselected patients receiving tamoxifen (78.4%) and similar to that of patients receiving an AI (83.2%). Two-way sensitivity analyses demonstrated the robustness of the results.

**Conclusions:**

Our modeling analyses indicate that, among EM patients, the DFS/EFS outcome of patients receiving tamoxifen is similar to that of patients receiving an AI. Further prospective clinical trials are needed to evaluate the value of the *CYP2D6* genotype in the selection of endocrine therapy.

## Introduction

Adjuvant tamoxifen is a fundamental systemic therapy for patients with hormone receptor-positive breast cancer [1]. Two minor but extremely active metabolites of tamoxifen, 4-hydroxytamoxifen and 4-hydroxy-N-desmethyltamoxifen (endoxifen), have been indicated to be predominantly catalyzed by cytochrome P450 2D6 (CYP2D6) [2]. The plasma concentrations of endoxifen could be affected by the genotypes coding for the CYP2D6 enzyme [3]. Therefore, the clinical efficacy of tamoxifen may vary according to *CYP2D6* genotypes. The *CYP2D6* gene is highly polymorphic, and its phenotypes are usually categorized into four groups: poor metabolizer (PM), intermediate metabolizer (IM), extensive metabolizer (EM), and ultra-rapid metabolizer (UM) [4]–[6].

In recent years, the role of tamoxifen in postmenopausal breast cancer patients has been challenged by aromatase inhibitors (AIs) [7], [8], which have been considered to be an optimal adjuvant endocrine treatment for postmenopausal women with hormone receptor-positive breast cancer [9]–[11]. However, there is concern that the up-front use of AIs does not result in an improvement in the overall survival compared with tamoxifen. Moreover, AIs do not always represent the ideal therapy for postmenopausal women because of the more common and severe musculoskeletal complaints and the higher risk of osteoporosis [12], [13]. In addition, AIs are expensive. Although some investigators [14], [15] have asserted that AIs are more cost-effective in an adjuvant setting, the cost of AIs varies vastly among countries (e.g., in China, anastrozole CNY1400/month vs. tamoxifen CNY30/month; in Locker's report [14]: anastrozole $6.56/day vs. tamoxifen $1.33/day). Considering the absolute 5-year disease-free survival (DFS) difference between tamoxifen and AIs is 2–4% [7], [8], the ability to select the patients who are likely to have a better response to AIs relative to tamoxifen is critical. Some studies have shown that women homozygous for the CYP2D6*4 allele (the most common PM allele in Caucasians [16]) had significantly lower plasma endoxifen concentrations [3] and worse clinical outcomes than women heterozygous or homozygous for the common alleles when given tamoxifen [17]–[21]; however, not all epidemiologic evidence supports this observation [22]–[24].

Recently, Punglia et al. [25] established a model using data from Goetz's study [17] to estimate whether women with wild-type *CYP2D6* have superior DFS outcomes if they receive tamoxifen rather than an AI. By applying the model, Punglia et al. proposed that women with wild-type *CYP2D6* actually had a similar or lower rate of relapse when treated with tamoxifen compared with an AI. Given that approximately 70% of women harbor wild-type *CYP2D6*, the role of *CYP2D6* genotype testing may be critical for selecting the optimal adjuvant endocrine treatment for postmenopausal patients [25].

Before the real-world application of the model developed by Punglia et al. [25], some questions should be resolved. First, the model is based on a relatively small sample size (*n* = 223) from a prospective cohort of the US North Central Cancer Treatment Group (NCCTG) 89-30-52 trial. Therefore, the representation of those data is limited and questionable. Of note, a recent JAMA article [26] reported updated results by combining data from a retrospective German breast cancer cohort with the original data from the NCCTG 89-30-52 trial cohort, resulting in a larger sample size (*n* = 1,325) and a median follow-up time of 6.3 years. It is necessary to reevaluate the old model using new and more convincing data. In addition, the model developed by Punglia et al. solely focuses on the CYP2D6*4 allele. Although *4 is the most frequent PM allele in Caucasians, other PM/IM alleles, including *3, *5, *6, *10, and *41, are also relatively common [6], [16]. Thus, a new model based on multiple genotypes should be proposed.

The aim of the present study was to establish a model using multiple-genotype-based data from a large sample size study to better determine whether treatment with an AI or tamoxifen is the optimal adjuvant endocrine therapeutic choice for postmenopausal patients harboring wild-type CYP2D6 enzymatic activity. The survival data for modeling was obtained from the Breast International Group (BIG) 1-98 trial [8]. In addition, our results were further validated using survival data in another large trial Arimidex, Tamoxifen, Alone or in Combination (ATAC) [7].

## Materials and Methods

### Data collection and assumptions

#### Annual hazard rates

Model estimates for relapse probabilities by initial treatment (AI or tamoxifen) were derived from the annual hazard rates in BIG 1-98 and ATAC trials (**Table 1**). Annual hazard rates in the BIG 1-98 trial was retrieved as previously described [25]. Annual hazard rates in the ATAC trial were obtained by measuring the annual hazard curves for time to recurrence in the article using the Measure Tool of Adobe Acrobat 7.0 Professional (Adobe Systems Inc., San Jose, California). A hazard rate at the middle of a given year was measured to represent the annual hazard rate. We assumed that the relapse probabilities during a given year were constant. We also assumed that neither the hazard rate for relapse on AIs nor the tumor characteristics were affected by metabolizer status and *CYP2D6* genotype.

**Table 1:** Model parameters definition.

Parameter			Reference
***Annual hazard rates for DFS (BIG 1-98)***			BIG 1-98 [8]
***Year***	***With an AI***	***With tamoxifen***	
**Year 0–1**	0.0243	0.0264	
**Year 1–2**	0.0268	0.0460	
**Year 2–3**	0.0415	0.0469	
**Year 3–4**	0.0414	0.0481	
**Year 4–5**	0.0401	0.0397	
***Annual hazard rates for EFS (ATAC)***			
**Year 0–1**	0.0127	0.0170	ATAC [7]
**Year 1–2**	0.0212	0.0303	
**Year 2–3**	0.0229	0.0291	
**Year 3–4**	0.0212	0.0269	
**Year 4–5**	0.0200	0.0283	
**Year 5–6**	0.0200	0.0285	
**Year 6–7**	0.0209	0.0264	
**Year 7–8**	0.0217	0.0242	
**Year 8–9**	0.0203	0.0279	
***Data sources for modeling***			Schroth et al. [26]
Metabolizer status	Genotype		HR
Extensive metabolizer(EM, 46.0%)	EM have normal enzyme function and were characterized by the absence of PM and IM alleles, but including UM.		1.0 (reference)
Decreased metabolizer(DM, 54%) = hetEM/IM (48.1%) + PM (5.9%)	IM have reduced enzyme activity and carry *10 and *41 alleles either homozygous or in combination with a PM allele. Heterozygous carriers of PM or IM alleles (hetEM) was combined with IM to define a group associated with intermediate impairment of CYP2D6 activity (hetEM/IM). PM indicates homozygous or compound heterozygous for *3, *4, or *5 alleles		1.29 (1.03–1.61) for DFS1.33 (1.06–1.68) for EFS

#### Data sources for CYP2D6 multiple-genotype-based modeling analyses

To construct a multiple-genotype-based model, we collected the data from the study by Schroth et al. [26], in which the investigators tested the ability of germline genetic variants in the *CYP2D6* gene to predict tamoxifen treatment outcomes in non-randomized postmenopausal hormone receptor-positive patients. In their study, Schroth et al. successfully genotyped the *3, *4, *5, *10, and *41 alleles and simultaneously analyzed gene duplication. The investigators divided the *CYP2D6* metabolizer statuses into extensive metabolizer (EM, denoting patients with two functional alleles, including those with ultra-rapid metabolism), heterozygote-extensive/intermediate metabolizer (hetEM/IM, denoting patients with intermediate or one poor metabolism allele), and poor metabolizer (PM, denoting patients homozygous for poor metabolism alleles) based on the genotypes of the combined *3, *4, *5, *10, and *41 alleles (**Table 1** and **Table 2**). The decreased metabolizer (DM) was defined as the combined PM and hetEM/IM groups. In our modeling analysis, we classified the *CYP2D6* metabolizer status into the EM group (46%) and the DM group (hetEM+IM+PM, 54%).

**Table 2:** Comparisons of two models.

Characteristics	Model	
	Punglia's	Our
Patients number for modeling	190	1,325
Alleles in modeling	*4	*3, *4, *5, *10, *41
HR of risk genotype (95%CI)	HR._*4/*4_ = 1.86 (0.91–3.82) for DFS [17]	*HR.*_DM_ = 1.29 (1.03–1.61) for DFS*HR.*_DM_ = 1.33 (1.06–1.68) for EFS [26]
Number (frequency) of PM, IM (hetEM/IM), EM(homEM)	13 (6.8%), 40 (21.1%), 137 (72.1%)	79 (5.9%), 637 (48.1%), 609 (46.0%)#
Parameters for modeling	4 (HR._*4/*4_, *Eff.*_wt/*4_, f._*4/*4_, *f.*_wt/4_)	2 (*HR.*_DM_, *f.*_DM_)

DM, PM, IM, and EM denote decreased metabolizer, poor metabolizer, intermediate metabolizer, and extensive metabolizer, respectively. het. heterozygous; hom. homozygous; DFS, disease-free survival; EFS, event-free survival; f, frequency; HR, hazard ratio.

# frequency of DM (indicating PM+IM+hetEM) is 54%.

#### Definition of survival end points

In this study, the definitions of survival end points were in accordance with the description in the BIG 1-98 [8] and ATAC trials [7], respectively. Survival simulation was performed using the annual hazard rates that were also derived from these two trials. For the BIG 1-98 trial [8], the annual hazard rate was for “disease” (disease-free survival [DFS] as the survival end point), which was defined as an invasive recurrence in local, regional, or distant sites; a new invasive breast cancer in the contralateral breast; any second (non-breast) malignancy; or death from any cause. For the ATAC trial [7], the annual hazard rate was for “event” (event-free survival [EFS] as the survival end point), which was defined as a local, regional, or distant recurrence; a new primary breast cancer (including new contralateral tumors); or death from breast cancer-specific causes. The hazard ratio (HR) of a “recurrence event” and the HR of “disease” for DM patients were 1.33 (95% confidence interval [CI]: 1.06–1.68) and 1.29 (95% CI: 1.03–1.61) relative to EM patients, respectively [26]. Unlike the model of Punglia et al. [25] with three levels of metabolizer status, our model had only two levels. Such a classification simplified the modeling procedure (**Table 2**).

### Markov model design

Markov decision models were developed using the TreeAge.Pro 2009 software (TreeAge Software, Williamstown, MA) to simulate the clinical histories of hypothetical cohorts of postmenopausal women with hormone receptor-positive breast cancer as previously described [25]. The Markov model simulated the transition between two health states, from a status of being well without any evidence of breast cancer events (event-free or disease-free) to an event status. The basic procedure of model establishment was as previously described [25]. We ran the model 60 times (by monthly cycle) to calculate the 5-year DFS probability and ran the model 108 times (by monthly cycle) to calculate the 9-year EFS probability. Using the survival data from the BIG 1-98 trial, our model calculated a 5-year DFS of 83.7% for those receiving an AI (letrozole) and 80.9% for those receiving tamoxifen. These data were consistent with the real 5-year DFS outcomes reported in the BIG 1-98 trail (84.0% and 81.1% for letrozole and tamoxifen, respectively). Using the data from the ATAC trial, our model obtained a 9-year EFS of 83.2% for those receiving an AI (anastrozole) and 78.4% for those receiving tamoxifen. These simulated results were also similar to the real 9-year EFS outcomes reported in the ATAC trail (83.0% and 78.2% for anastrozole and tamoxifen, respectively).

### Sensitivity analyses

We performed sensitivity analyses using the TreeAge.Pro 2009 software (TreeAge Software, Williamstown, MA). In our model, there were two parameters: hazard ratio of DM (*HR.*_DM_) and its frequency (*f.*_DM_). We performed the two-way sensitivity analysis by simultaneously varying *HR.*_DM_ and *f.*_DM_. *HR.*_DM_ varied from 1.05 to 3.05 according to the reported 95% CI of EFS (95% CI: 1.06–1.68 [26] and 95% CI: 1.10 to 3.25 [20]), whereas *f.*_DM_ varied according to its assumed extreme values by the following formula: 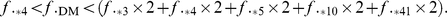



The above formula was determined according to the definition of a DM in the original paper [26]. By reviewing the allelic frequency data of *3/*4/*5/*10/*41 in previous reports [4], [27], we assumed the lower limit and upper limit of *f.*_DM_ were approximately 20% and 80%, respectively. Note that the real interval of *f.*_DM_ should be much narrower.

## Results

### The multiple-genotype-based modeling analysis indicated a similar role of TAM to AI in postmenopausal breast cancer women with wild-type *CYP2D6* using BIG 1-98 survival data

**Figure 1:**
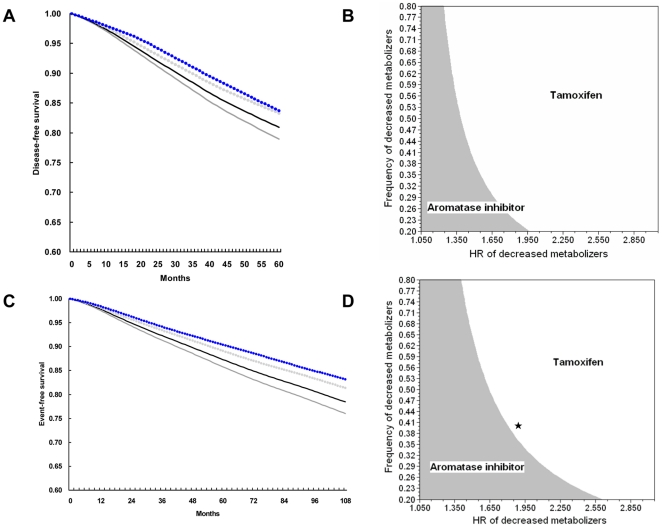
Survival simulation and two-way sensitivity analyses of the *CYP2D6* multiple-genotype-based model. (**A**) Simulated 5-year disease-free survival (DFS) curves for an unselected population and each metabolizer-based subgroup using the hazard rate data from the BIG 1-98 trial. We used an *HR.*_DM_ of 1.29 and an *f.*_DM_ of 0.54. The dotted blue line represents the AI strategy in the unselected population. The black line represents the tamoxifen strategy in the unselected population. The tamoxifen treatment in EM patients is shown as a dotted gray line, and DM patients are represented by the dark-gray line. The simulated 5-year DFS for EM patients, DM patients, and unselected women treated with tamoxifen and unselected women treated with an AI were 83.3%, 77.0%, 80.9%, and 83.7%, respectively. (**B**) Two-way sensitivity analysis for EM patients by varying *HR.*_DM_ and *f.*_DM_ using the hazard rate data from the BIG 1-98 trial. *HR.*_DM_ is plotted on the x-axis, and *f.*_DM_ is plotted on the y-axis. The gray area represents the combinations of *HR.*_DM_ and *f.*_DM_ for which an AI optimizes the 5-year DFS for EM patients, and the white area represents those for which tamoxifen optimizes the 5-year DFS for EM patients. (**C**) Simulated 9-year event-free survival (EFS) curves for an unselected population and each metabolizer-based subgroup using the hazard rate data from the ATAC trial. We used an *HR.*_DM_ of 1.33 and an *f.*_DM_ of 0.54. The dotted blue line represents the AI strategy in the unselected population. The black line represents the tamoxifen strategy in the unselected population. The tamoxifen treatment in EM patients is shown as a dotted gray line, and DM patients are represented by the dark-gray line. The simulated 9-year EFS for EM patients, DM patients, and unselected women treated with tamoxifen and unselected women treated with an AI were 81.4%, 76.0%, 78.4%, and 83.2%, respectively. (**D**) Two-way sensitivity analysis for EM patients by varying *HR.*_DM_ and *f.*_DM_ using the hazard rate data from the ATAC trial. The pentalpha maker corresponds to another previously published estimate by Schroth et al. (*f.*_DM_  = 40%, *HR.*_DM_  = 1.89, 95% CI: 1.10–3.25) [20].

We used the multiple-genotype-based model to examine 5-year DFS by CYP2D6 metabolizer status. In the base case analysis, we used an *HR.*_DM_ (HR for “disease” among DM patients receiving tamoxifen) of 1.29 and an *f.*_DM_ of 0.54. The simulated 5-year DFS of tamoxifen-treated EM patients was 83.3%, which was similar to that for pharmacogenetically unselected patients treated with an AI (letrozole) of 83.7% [8]. **Figure 1A** shows the DFS curves for all patients treated with an AI or tamoxifen as well as for tamoxifen-treated subpopulations divided by metabolizer statuses. Notably, the simulated survival curves were derived from assumed data rather than truly observed data. Our findings, based on the larger sample size study [26], were consistent with the results from another model proposed by Punglia et al. [25].

We next investigated the robustness of the findings across a range of assumptions for *HR.*_DM_ and *f.*_DM_ using a two-way sensitivity analysis by simultaneously varying *HR.*_DM_ and *f.*_DM_. The results shown in **Figure 1B** are from EM patients only. Each point on this figure can be described by an (x, y) coordinate. The x-axis plots *HR.*_DM_, whereas the y-axis plots *f.*_DM_. Each (x, y) point represents a unique combination. The white area therefore indicates the combinations of *HR.*_DM_ and *f.*_DM_ parameters, for which tamoxifen optimizes the 5-year DFS in EM patients, and the grey area depicts those for which an AI optimizes the 5-year DFS in EM patients. By this analysis, we observed that when *HR.*_DM_ >1.95, almost all EM patients would benefit more from tamoxifen than from an AI, whereas if *HR.*_DM_ <1.25, the use of tamoxifen in EM patients may be less beneficial than an AI. When *HR.*_DM_ is between 1.25 and 1.95, the choice of endocrine therapy depended on the value of *f.*_DM_. A higher *f.*_DM_ represented a higher possibility of benefiting from tamoxifen.

### The comparable role of tamoxifen with AIs in women with breast cancer who presented with wild-type *CYP2D6* is successfully replicated using ATAC survival data

We further tested our model using the survival data from another large randomized clinical trial, the ATAC [7]. In the base case analysis, we used an *HR.*_DM_ of 1.33 for EFS and an *f.*_DM_ of 54%. The 9-year EFS of tamoxifen-treated EM patients was 81.4%, which is higher than that of genotypically unselected patients receiving tamoxifen (78.4%) and similar to that of patients receiving an AI (anastrozole) (83.2%). **Figure 1C** displays the EFS curves for all genotypically unselected patients treated with an AI or tamoxifen and for genotypically selected patients treated with tamoxifen.

Likewise, we performed a two-way sensitivity analysis by varying *HR.*_DM_ and *f.*_DM_ (**Figure 1D**). **Table 3** displays the results of the sensitivity analysis in a digital form. We found that when *HR.*_DM_ >1.5, a relative higher *f.*_DM_ (≥60%) would warrant the survival benefits from tamoxifen in EM patients. Once *HR.*_DM_ ≥2.5, the EM women would absolutely benefit more from tamoxifen than from an AI. Conversely, if *HR.*_DM_ <1.3, an AI would likely be a better option for EM patients.

**Table 3:** The two-way sensitivity analysis of the model using ATAC hazard rate data.

*f.*_DM (%)_	*HR.*_DM_				
	1.1	1.5	2.0	2.5	3.0
**20**	79.0	80.3	81.8	**83.1**	**84.2**
**30**	79.1	81.1	**83.1**	**84.7**	**86.0**
**40**	79.3	81.8	**84.2**	**86.0**	**87.5**
**50**	79.5	82.5	**85.2**	**87.1**	**88.6**
**60**	79.7	**83.1**	**86.0**	**88.1**	**89.6**
**70**	79.8	**83.6**	**86.8**	**88.9**	**90.4**
**80**	80.0	**84.2**	**87.5**	**89.6**	**91.1**

The 9-year event-free survival (EFS) for EM patients receiving tamoxifen is shown by varying *HR.*_DM_ and *f.*_DM_. EFS is expressed as a percentage. EFS outcomes which are equal to or greater than those for patients receiving an AI (≥83.0%, data from ATAC trial) are shown in bold font. The graphical form of this table is shown in **Figure 1D**.

## Discussion

Epidemiological evidence from retrospective studies indicates an association between *CYP2D6* variations and altered tamoxifen response in a range of therapeutic settings such as metastatic breast cancer [28], cancer prevention [29], and adjuvant therapy [17], [18], [20], [21]. In the adjuvant setting, some studies have suggested that PM/IM patients might gain insufficient therapeutic benefits from tamoxifen and be at a higher risk of breast cancer relapse than EM patients. The present study is based on the assumption that the pharmacogenetics of tamoxifen biotransformation is associated with clinical outcomes. We constructed a *CYP2D6* multiple-genotype-based model using two convenient parameters, the HR of DM (*HR.*_DM_) and its frequency (*f.*_DM_). Our model is more feasible to perform and is likely more reliable than a single allele (*4)-based model. We also replicated the modeling outcome with survival data from another large trial, the ATAC trial. The validation results were consistent with the initial findings.

The current study strongly suggests that the adjuvant endocrine therapy should be tailored for an individual patient according to her multiple *CYP2D6* genotypes. This statement, however, may be premature. Only one side of the coin is being considered by looking at *CYP2D6* genotypes for tamoxifen metabolism, and there are many other factors involved in endocrine therapy response. For example, *CYP19* genotypes might modulate AI metabolism [30]; the alleles of *ABCC2* have been shown to have an additive effect on recurrence-free survival outcome of adjuvant tamoxifen therapy for breast cancer patients. Furthermore, the role of tumor characteristics was not considered in the present study. In one report, a composite index comprising of host *CYP2D6* polymorphisms along with tumor homeobox-13 and interleukin-17B receptor ratio could accurately predict tamoxifen sensitivity than either alone [31]. To date, no prospective trial has been conducted to test the hypothesis that *CYP2D6* pharmacogenetic testing can predict tamoxifen response. The available evidence does support the launch of a clinical trial to scrutinize the value of *CYP2D6* genotypes in endocrine therapy selection. In fact, AIs are not always appropriate for “all” postmenopausal patients in clinic practice due to their common toxicity resulting in arthralgias and/or bone pain as well as their higher cost [12]. Despite the absence of primary evidence from prospective trials, it seems reasonable at present the use clinical judgment to utilize *CYP2D6* testing under certain conditions; however, its use should be confined [32].

Our modeling analyses have unavoidable limitations. First, in addition to CYP2D6, other CYP isoforms such as CYP3A4/5, CYP2C19, CYP2C9, and CYP2B6 appear to play less important but somewhat unique roles in tamoxifen metabolism [3]. The present multiple-genotype-based model still fails to integrate them together because of insufficient resource data. Second, our model is only applicable to Caucasian women, as the distribution and frequency of *CYP2D6* genotypes in Asians and Africans are somewhat different [16]. Third, tamoxifen metabolism can be mediated by pharmacologic inhibitors of CYP2D6 [3], [33]. Certain selective serotonin reuptake inhibitors (SSRIs) are potent inhibitors of CYP2D6, and co-administration of SSRIs would negatively alter the efficacy of tamoxifen [3], [33]. Our model did not consider the effect of comedication of CYP2D6 inhibitors on survival outcomes.

Taken together, the current evidence is still not strong enough to warrant an ethical obligation for physicians to inform postmenopausal patients with hormone receptor-positive breast cancer about the *CYP2D6* genotype testing when deciding between tamoxifen and an AI in an adjuvant setting. The findings in the present study, however, strongly suggest that adjuvant endocrine therapy should be tailored to each individual patient according to her genetic information, especially for those women who are concerned about the toxicity or cost of AIs.
